# Implementing a Tiered Genetic Testing Strategy for Muscular Dystrophies in Morocco: From Targeted Assays to Exome Sequencing

**DOI:** 10.1002/mgg3.70192

**Published:** 2026-02-25

**Authors:** Yasmina Rahmuni, Ilham Ratbi, Jaber Lyahyai, Imane Cherkaoui Jaouad, Ourayna Batta, Aziza Sbiti, Maryem Sahli, Omar Askander, Zineb Rchiad, Abdelaziz Sefiani

**Affiliations:** ^1^ Research Team in Genomics and Molecular Epidemiology of Genetic Diseases, GENOPATH Center, Faculty of Medicine and Pharmacy University Mohammed V of Rabat Rabat Morocco; ^2^ Department of Medical Genetics National Institute of Health Rabat Morocco; ^3^ Unity of Medical Genetics, Children's Hospital, Ibn Sina University Hospital Center, Faculty of Medicine and Pharmacy Mohammed V University of Rabat Rabat Morocco; ^4^ Mohammed VI Center for Research and Innovation (CM6RI) Rabat Morocco; ^5^ Genomics Platform, UM6P Corelab University Mohammed VI Polytechnic Benguerir Morocco

**Keywords:** customized panel, dystrophinopathy, LGMD, muscular dystrophy, NGS, WES

## Abstract

**Introduction:**

Muscular dystrophies (MDs) are a heterogeneous group of inherited neuromuscular disorders. In Morocco, where consanguinity is common, recurrent variants have been reported; however, the overall molecular landscape remains underexplored.

**Methods:**

We analyzed 716 patients referred over 32 years for suspected limb‐girdle muscular dystrophy (LGMD) or dystrophinopathy using a stepwise approach. Multiplex PCR (MPCR) for *DMD* deletions and targeted Sanger sequencing of the *SGCG*:c.525delT variant were performed as first‐line tests. Unsolved patients underwent next‐generation sequencing (NGS), either via a customized gene panel or whole‐exome sequencing (WES).

**Results:**

Multiplex PCR for *DMD* deletions and targeted Sanger sequencing of the *SGCG*:c.525delT variant resolved nearly half of cases, demonstrating the efficiency of these cost‐effective first‐line tests. Unsolved patients underwent next‐generation sequencing (NGS), via a customized gene panel or whole‐exome sequencing (WES), though resource limitations prevented full coverage. Among tested cases, *SGCA*, *CAPN3*, and *FKRP* were the most frequently mutated genes after *DMD* and *SGCG*, with several novel or recurrent variants suggesting population‐specific alleles.

**Conclusions:**

These results highlight the genetic heterogeneity of MDs in Morocco and the value of combining targeted and broad genomic approaches, while underscoring the need to extend NGS to fully characterize unresolved patients.

## Graphical abstract

Targeted routine testing combined with NGS approaches enabled effective genetic diagnosis of muscular dystrophies in Moroccan patients, with nearly half of cases resolved by first‐line testing and additional diagnoses obtained through targeted sequencing and whole‐exome sequencing.

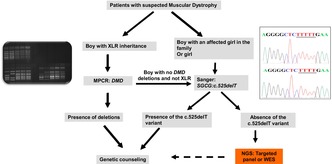

## 1 Introduction

Myopathies refer to a heterogeneous group of muscle disorders characterized by muscle fiber dysfunction, which may be genetic or acquired. Among these, muscular dystrophies (MD) represent a subgroup of genetic myopathies, histologically characterized by dystrophic changes visible on muscle biopsy. However, as biopsy is an invasive procedure, molecular biology technologies have become essential alternatives for accurate and less invasive diagnosis of these diseases (Gonzalez‐Quereda et al. 2020; Srivastava et al. 2020). MD encompasses a wide spectrum of clinical phenotypes, modes of inheritance, age of onset, and severity. The most common types include Duchenne/Becker muscular dystrophy (DMD/BMD), limb‐girdle muscular dystrophies (LGMD), congenital muscular dystrophies, and Emery–Dreifuss muscular dystrophy (EDMD). Although they share overlapping clinical features, each subtype is associated with specific genetic alterations and diagnostic approaches.

In resource‐limited settings, establishing an efficient and cost‐effective diagnostic strategy is essential for the management of neuromuscular disorders. Given the clinical and genetic heterogeneity of MD, a stepwise approach starting with targeted analyses based on clinical presentation and inheritance patterns and progressing to broader sequencing methods can help optimize diagnostic yield while minimizing costs. Moroccan population is characterized by a high rate of consanguinity, which increases the likelihood of autosomal recessive disorders and reinforces the need for tailored diagnostic strategies that take population‐specific genetic features into account (Jaouad et al. 2009).

In this study, we present the results of a diagnostic approach applied to a cohort of Moroccan patients referred to our department for suspected MD, including both DMD/BMD and LGMD during more than three decades. The cohort includes both retrospectively and prospectively analyzed cases. We describe a tiered diagnostic strategy consistently applied to all patients, beginning with first‐line targeted analyses, such as Sanger sequencing and multiplex polymerase chain reaction (MPCR), followed by next‐generation sequencing (NGS) gene panels or whole‐exome sequencing (WES) in unresolved cases. We also assess the diagnostic yield and relevance of each method within this specific population.

## 2 Materials and Methods

### 2.1 Patients and Study Design

This study enrolled 716 patients referred to the medical genetics department of the National Institute of Health in Rabat over a 32‐year period (1992–2024) for suspected muscular dystrophies. The cohort, including cases recruited retrospectively and prospectively from various regions of Morocco, represents one of the largest national collections of patients with suspected muscular dystrophies. Patients were initially referred for clinical suspicion of either LGMD or dystrophinopathy.

Most patients had markedly elevated serum creatine kinase (CK) levels and electromyography (EMG) findings consistent with a myogenic profile. In some cases, a positive family history was reported, including cases of consanguinity or recurrence of similar symptoms in siblings, reflecting the underlying genetic profile of the Moroccan population.

All patients were evaluated using a standardized, stepwise diagnostic strategy based on clinical presentation, inheritance pattern, and resource availability. Table 1 presents the main characteristics of the patients included in this study.

**TABLE 1:** Baseline characteristics of the study cohort (*n* = 716).

Characteristic	LGMD‐suspected (*n* = 266)	Dystrophinopathy‐suspected (*n* = 450)	Total cohort (*n* = 716)
Sex ratio (M/F)	112/154	450/0	562/154
Mean age at onset (years)	6.6 years old	5.53 years old	5.9 years old
Age range (years)	2–65 years old	2–24 years old	2–65 years old
Elevated CPK (%)	245/266 (92.1%)	450/450 (100%)	695/716 (97%)
Myopathic EMG (%)	143/266 (53.8%)	226/450 (50.2%)	269/716 (37.5%)
Positive family history (%)	55/266 (20.6%)	91/450 (20.2%)	146/716 (20.3%)
Consanguinity (%)	94/266 (35.3%)	124/450 (27.5%)	218/716 (30.4%)

Abbreviations: CPK, creatine phosphokinase; EMG, electromyography; LGMD, limb girdle muscular dystrophy; M/F, male/female.

### 2.2 Sample Collection and DNA Extraction

Peripheral blood samples were collected from all patients after obtaining informed consent, either directly from the patients or their legal guardians for minors. Genomic DNA was extracted using different methods depending on the period and available resources. Older samples underwent DNA extraction using the salting‐out method. Recent extractions were either performed manually using the PureLink TM Genomic DNA Mini Kit (Invitrogen—Thermo Fisher Scientific—USA), according to the manufacturer's recommendation, or semi‐automatically using the MagMax system. DNA concentration and quality were assessed for each sample using both the A260/280 ratio measured by a Nanodrop spectrophotometer (Fisher Scientific, Wilmington, DE) and for more precise quantification by a Qubit 3.0 Fluorometer with the Qubit dsDNA HS Assay Kit, especially before NGS analysis.

This study was approved by the Rabat Ethics Committee for Biomedical Research (Approval Number: CERB‐37–23).

### 2.3 Diagnostic Workflow

A multi‐level diagnostic strategy was implemented to ensure cost‐effectiveness and adaptability to contexts where resources are limited:

#### 2.3.1 First‐Line Tests Included

1. MPCR analysis of the *DMD* gene was performed in male patients with clinical features suggestive of dystrophinopathy and a pedigree consistent with X‐linked recessive transmission, in order to detect common exon deletions as previously reported by Sbiti et al. (Sbiti et al. 2002).

In total, 450 patients underwent MPCR testing.

2 Given its high prevalence in Moroccan and North African populations, targeted Sanger sequencing of exon 6 of the *SGCG* gene was carried out systematically to screen for the known pathogenic variant NM_000231.3(*SGCG*):c.525delT(p.Phe175LeufsTer20). This analysis was applied not only to patients with LGMD phenotypes suggestive of autosomal recessive transmission, but also to male patients who tested negative for *DMD* deletions by MPCR, as previously reported (El Kerch et al. 2014). Sanger sequencing was performed using the BigDye Terminator v3.1 Cycle Sequencing Kit (Applied Biosystems, Life Technologies), then purified using the Sephadex method (Sigma‐Aldrich Co. LLC). Electrophoresis and data collection were performed on an ABI 3500 genetic analyzer (Applied Biosystems, Thermo Fisher Scientific). The sequences obtained were aligned to the GRCh37/hg19 reference genome using Mutation Surveyor software (SoftGenetics).

This assay was applied to 299 patients.

#### 2.3.2 Second‐Line Test Was Performed on Cases With Negative Initial Results

**FIGURE 1:**
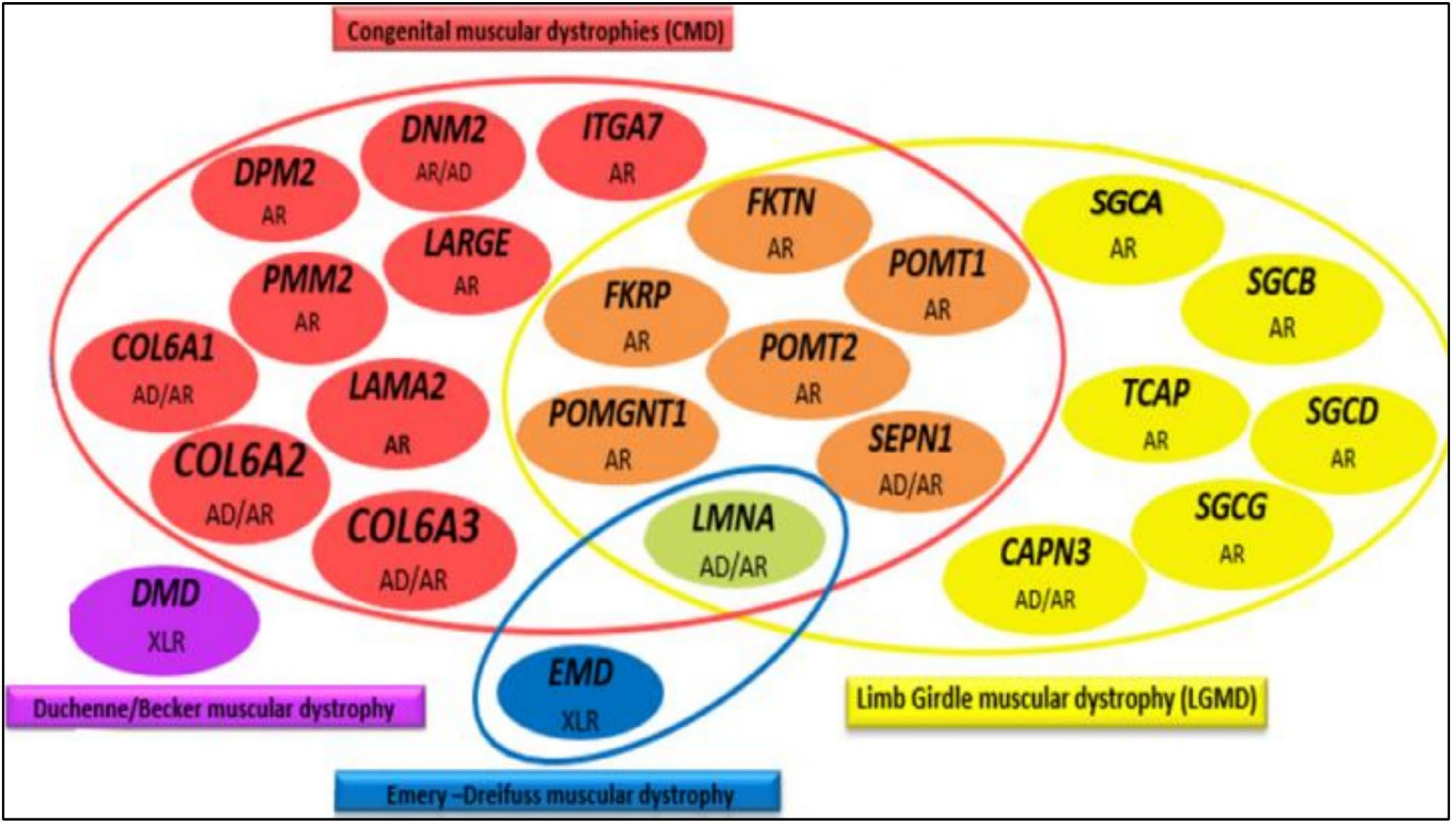
Customized gene panel used for the molecular diagnosis of muscular dystrophies and related myopathies. The panel encompasses the major genes implicated in muscular dystrophies and related myopathies, including congenital muscular dystrophies (CMD, red/orange), limb‐girdle muscular dystrophies (LGMD, yellow), Duchenne/Becker muscular dystrophy (DMD/BMD, purple), and Emery–Dreifuss muscular dystrophy (EDMD, blue). Some genes, such as *FKRP* and *LMNA*, are associated with more than one clinical phenotype. The inheritance pattern of each gene is indicated. AD, autosomal dominant; AR, autosomal recessive; XLR, X‐linked recessive.

1. A customized NGS gene panel targeting genes involved in rare diseases was performed using the AmpliSeq On‐Demand Primer Panel (Thermo Fisher Scientific), which consists of two primer pools. Library preparation and chip loading were performed using the Ion Chef system, and sequencing was completed on the Ion S5 platform, all in accordance with the kit instructions. Among the targeted genes, 24 were specifically associated with muscular dystrophies and related myopathies (Figure 1).

A total of 28 patients were analyzed using this customized panel.

2 WES was initially used as a third‐tier approach for patients who remained undiagnosed after first‐line and panel‐based testing. Over time, given the high cost and limited yield of sequential testing in certain unsolved cases, WES was also applied directly for some patients without prior gene panel analysis. This shift allowed us to optimize time and reduce overall costs by skipping the NGS panel in certain unresolved cases and moving directly to WES.

Overall, 42 patients underwent WES analysis.

### 2.4 Illumina DNA Prep With Exome 2.5 Enrichment Protocol

Library preparation used Illumina DNA Prep with Exome 2.5 Enrichment. Briefly, bead‐linked transposomes (eBLT) carried out on‐bead tagmentation to fragment gDNA and append adapters, after which a limited‐cycle PCR added unique dual indexes. A double‐sided cleanup with Illumina Purification Beads generated normalized pre‐enrichment libraries (inputs ≥ 50 ng do not require pre‐hybridization quantification; inputs 10–49 ng require quantification and adjusted PCR cycles). Pre‐enrichment libraries were qualified (modal fragment size ~300–400 bp), then pooled (up to 12‐plex) and hybridized with the 120‐bp, biotinylated Twist for Illumina Exome 2.5 probes at 62°C. Targeted hybrids were captured on streptavidin magnetic beads, washed, eluted, and amplified to yield exome‐enriched pools, which underwent a final bead cleanup and QC (Agilent TapeStation and Qubit) before sequencing on an Illumina platform. This workflow provides high‐quality, human whole‐exome libraries with streamlined normalization and robust compatibility with standard automation.

### 2.5 Data Analysis

Sequencing data obtained from targeted Sanger sequencing, gene panels, or WES were analyzed using validated bioinformatics pipelines specific to each platform.

For Sanger sequencing, electropherograms were interpreted using Sequencing Analysis Software version 7.0 (Applied Biosystems, Thermo Fisher Scientific). For the targeted gene panel, variant calling and annotation were performed using Ion Reporter Software (Thermo Fisher Scientific). All variants were visually verified using the Integrative Genomics Viewer (IGV) v2.2, Broad Institute, and described according to the nomenclature guidelines of the Human Genome Variation Society (HGVS; http://www.hgvs.org). For WES data, VCF files received from the sequencing provider were further analyzed using the Franklin platform (Genoox, https://franklin.genoox.com), which enabled clinical annotation and variant classification. The interpretation of identified variants was conducted following the American College of Medical Genetics and Genomics (ACMG) guidelines.

## 3 Results

Among the 716 patients included in this study, 450 were initially screened for recurrent deletions in the *DMD* gene using multiplex PCR, of whom 202 tested positive (44.9%). In parallel, 299 patients were tested for the recurrent mutation in the *SGCG* gene, identifying 136 positive cases (45.5%). Notably, 72 boys who were negative for *DMD* deletions underwent additional testing for the recurrent *SGCG* mutation, and 33 of them were positive (already included in the *SGCG*‐positive count above), indicating no overlap of positive results between the two assays. Overall, these two targeted approaches established a molecular diagnosis in 48% of patients in the entire cohort.

**FIGURE 2:**
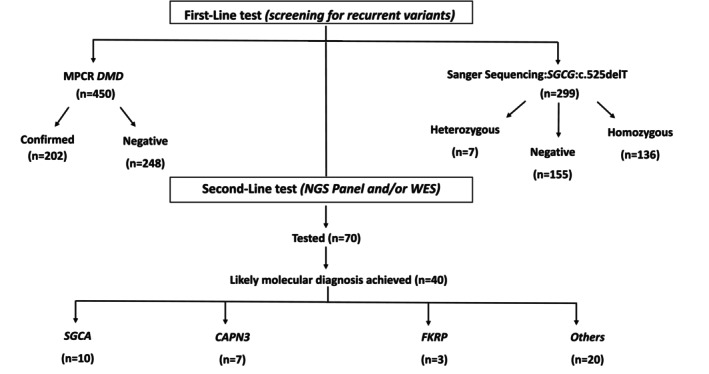
Flowchart of first and second‐line genetic testing. First‐line testing included MPCR of the *DMD* gene (*n* = 450) and targeted Sanger sequencing of the recurrent *SGCG*:c.525delT mutation (*n* = 299). Unsolved cases subsequently underwent second‐line testing using NGS panel and/or WES (*n* = 70), which led to a molecular diagnosis in 40 patients, mainly involving *SGCA*, *CAPN3*, *FKRP*, and other genes.

Beyond these two targeted assays, NGS approaches, including gene panels and WES, were performed in a subset of patients to broaden the molecular investigation. Among the 70 patients who underwent NGS testing, a likely molecular diagnosis was achieved in 40 cases, including those with pathogenic or likely pathogenic variants, as well as some cases with variants of uncertain significance (VUS). The results revealed a heterogeneous distribution of causative genes (Figure 2). The most frequently implicated gene was *SGCA*, mutated in 10 patients (14.3%), followed by *CAPN3* in 7 patients (10%), and *FKRP* in 3 patients (4.3%). Variants in 20 other genes were identified in the remaining cases, representing 50% of the diagnosed patients and 28.5% of the total NGS cohort. This group included genes associated with neuromuscular disorders (such as *COL6A1*, *COL6A2*, *SGCB*, *SGCD*, *DYSF*, *EMD*, and *LMNA*), as well as other muscular dystrophies and metabolic myopathies (e.g., *GAA*).

## 4 Discussion

**FIGURE 3:**
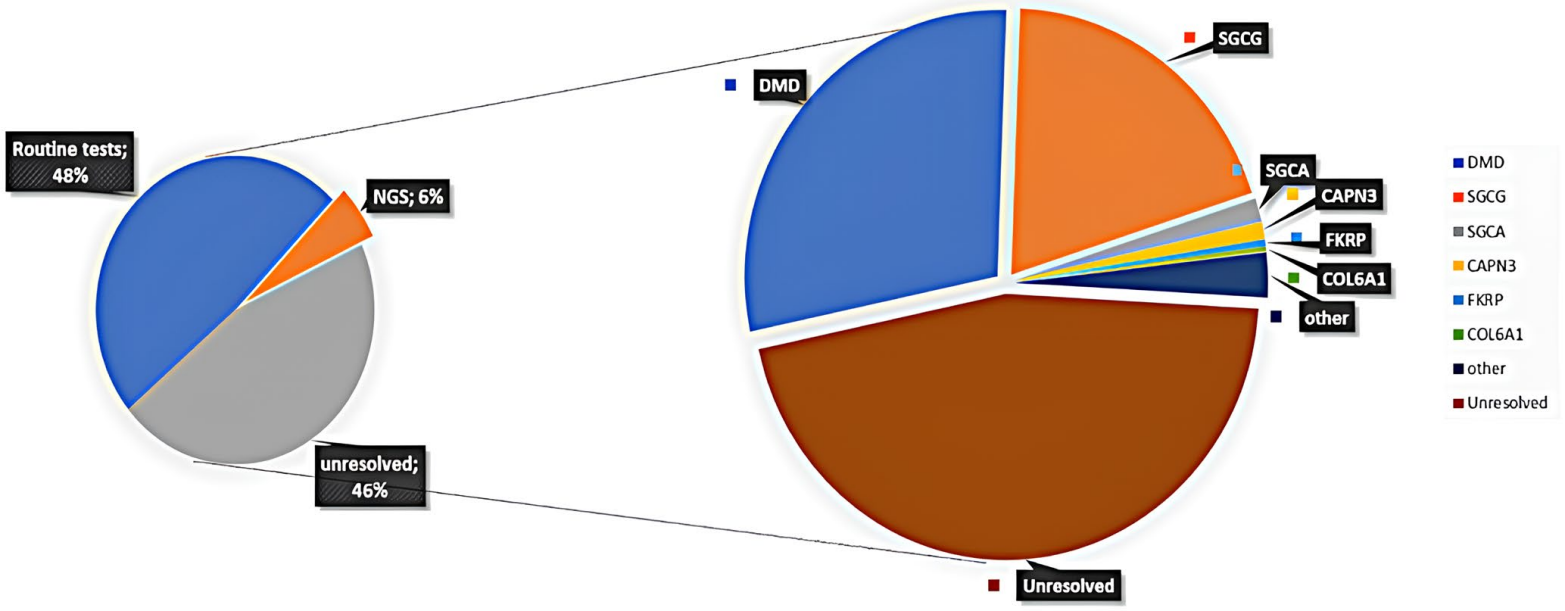
Overall diagnostic resolution and gene distribution in patients with suspected muscular dystrophies. The pie chart on the left shows the proportion of cases resolved by routine testing (MPCR and targeted Sanger sequencing) and by NGS approaches, as well as unresolved cases. The pie chart on the right shows the distribution of molecular diagnoses among different genes, with major contributions from *DMD*, *SGCG*, *SGCA*, *CAPN3*, and *FKRP*.

Muscular dystrophies represent a genetically and clinically heterogeneous group of disorders, and their molecular diagnosis remains a challenge, particularly in underrepresented populations. In our study, we adopted a stepwise strategy: as a first‐line test, recurrent deletions in the *DMD* gene were excluded using multiplex PCR, and the recurrent c.525delT mutation in the *SGCG* gene was systematically screened. Patients without these common alterations were subsequently investigated using a second‐line approach, either through targeted gene panels or whole‐exome sequencing (WES). This strategy allowed us to identify both previously reported and novel variants across several disease‐causing genes. Overall, our findings expand the mutational spectrum of muscular dystrophies in North Africa and emphasize the importance of integrating clinical, histopathological, and molecular data to refine diagnoses. Figure 3 summarizes the overall distribution of molecular diagnoses obtained through routine assays and NGS, as well as the proportion of unresolved cases.

In the following sections, we discuss our results according to the main categories of disorders identified in our cohort.

### 4.1 Dystrophinopathies

Dystrophinopathies, encompassing DMD and BMD, remain the most frequent forms of muscular dystrophy in children. In our cohort, 450 patients clinically suspected of DMD/BMD were screened by MPCR, a method widely used for its accessibility and efficiency in detecting the most common deletions within the DMD gene. Deletions were identified in 202 patients (≈44.9%), which is consistent with reported frequencies in the literature, where deletions account for approximately 60%–65% of *DMD* mutations (Singh et al. 2006). This 44.9% rate is very similar to that previously reported by our team in 2020, confirming the reproducibility of multiplex PCR results in our population (El Kadiri et al. 2020). Among patients who tested negative by MPCR, seven were further analyzed using a targeted NGS gene panel, leading to the identification of six novel variants, already reported in a previous study by our department (El Kadiri et al. 2020). These findings reinforce the utility of complementary molecular approaches to detect point mutations and small indels that escape PCR‐based methods.

### 4.2 LGMD

Given the well‐established founder effect of the c.525delT (p.Phe175LeufsTer20) pathogenic variant in the *SGCG* gene in North African populations, particularly in Morocco, targeted screening was performed in 299 patients clinically suspected of having LGMD. This recurrent mutation, previously reported by our team, has an estimated prevalence of 1 in 20,492 in the Moroccan population (El Kerch et al. 2014). Among the tested individuals, 136 (45.5%) were homozygous for the variant, 7 (2.3%) were heterozygous carriers, and 155 (51.8%) were negative. These findings confirm the high frequency of this founder mutation and support its inclusion as a first‐line, population‐specific screening tool in the molecular diagnosis of LGMD in endemic regions. Importantly, the two routine targeted tests, together, allowed a molecular diagnosis in nearly half of the patients, demonstrating that these assays remain an effective and practical first‐line screening strategy.

Considering the limited availability and the fact that NGS is not yet covered by healthcare, NGS, although available locally, is not routinely used as a diagnostic tool. Consequently, only 28 patients suspected of having muscular dystrophy were able to get targeted genetic testing using NGS. The results were both heterogeneous and instructive, revealing pathogenic or likely pathogenic variants in several genes. Among the patients tested by targeted gene panel sequencing after a negative result for the recurrent *SGCG* variant, the most frequently mutated gene was *SGCA*, with pathogenic or likely pathogenic variants identified in seven individuals. These findings highlight the genetic heterogeneity of LGMD in our cohort and suggest that LGMD R3 may be more prevalent than previously thought when the recurrent *SGCG* mutation is excluded.

#### 4.2.1 Recurrent Molecular Findings in NGS Results: Focus on SGCA, CAPN3, and FKRP

Among the patients who underwent NGS‐based testing (either by targeted gene panel or WES), the most frequently mutated genes were *SGCA*, *CAPN3*, and *FKRP* (Table 2). These findings are particularly noteworthy, as they were observed in multiple unrelated families, suggesting either a high prevalence of certain LGMD subtypes in the Moroccan population or the presence of possible founder mutations. The recurrence of pathogenic or likely pathogenic variants in these genes highlights their significant contribution to the molecular landscape of LGMDs in our cohort and supports the consideration of these genes as high‐priority candidates in diagnostic strategies. In‐depth discussion of these results is provided below.

**TABLE 2:** Genes with recurrent variants detected by NGS.

Gene	Variant (cDNA)	Protein change	Location	Allele count	Prediction (ACMG)	N/R	dbSNP ID	Detection method	Phenotype
*SGCA* *n* = 10 (20 alleles)	c.411dup	p.Phe138ValfsTer53	Exon 5	4/20	Likely pathogenic	N	NA	Targeted Panel	LGMD R3
	c.371T>C	p.Ile124Thr	Exon 4	2/10	Pathogenic	R	rs768814872	Targeted panel	
	c.161delT	p.Val54AlafsTer157	Exon 3	2/20	Pathogenic	R	rs886044516	Targeted panel	
	c.701delA	p.Asp234AlafsTer14	Exon 6	2/20	Pathogenic	R	rs2509123275	Targeted panel	
	c.956+1G>A	p.?	Intron 7	5/20	Likely pathogenic	R	rs2509125625	Targeted panel/WES	
	c.1A>G	p.Met1Val	Exon 1	2/20	Pathogenic	R	rs2509113878	Targeted panel	
	c.271G>A	p.Gly91Ser	Exon 3	2/20	Likely pathogenic	R	rs890921874	WES	
	c.401_408dup	p.Glu137ThrfsTer77	Exon 5	2/20	Likely pathogenic	N	NA	WES	
*CAPN3* *n* = 7 (14 alleles)	c.1714C>T	p.Arg572Trp	Exon 13	2/14	Pathogenic	R	rs863224959	Targeted panel	LGMD R1
	c.1448C>A	p.Ala483Asp	Exon 11	2/14	Pathogenic	R	rs781723572	WES	
	c.2129G>A	p.Gly710Glu	Exon 20	5/14	Likely pathogenic	N	NA	WES	
	c.1194‐9A>G	p.?	Intron 9	2/14	Pathogenic	R	rs374665929	WES	
	c.2242C>T	p.Arg748Ter	Exon 21	2/14	Pathogenic	R	rs768090444	WES	
*FKRP* *n* = 3 (6 alleles)	c.1364C>A	p.Ala455Asp	Exon 4	2/6	Pathogenic	R	rs28937903	Targeted panel	LGMD R9
	c.1327G>A	p.Glu443Lys	Exon 4	1/6	Likely pathogenic	R	rs1318972801	Targeted panel	
	c.1012G>T	p.Val338Leu	Exon 4	1/6	Pathogenic	R	rs1173430388	Targeted panel	
	c.1016G>C	p.Arg339Pro	Exon 4	2/6	Likely pathogenic	R	rs1450841129	WES	

Abbreviations: ACMG, American College of Medical Genetics and Genomics; LGMD R1, limb girdle muscular dystrophy recessive 1, calpain‐3 related; LGMD R3, limb girdle muscular dystrophy recessive 3, α‐sarcoglycan‐related; LGMD R9, limb girdle muscular dystrophy recessive 9, FKRP‐related; N, novel; NA, not available; R, reported; WES, whole‐exome sequencing.

#### 4.2.2 LGMD R3

Alpha‐sarcoglycanopathy (LGMD R3) is caused by pathogenic variants in the *SGCA* gene (OMIM #608099), located on chromosome 17q12–q21.3 and comprising 10 exons. Of these, exons 1–9 are coding, producing a major mRNA transcript of approximately 1200 bp, predominantly expressed in skeletal muscle, diaphragm, and cardiac muscle. As of August 2025, the Human Gene Mutation Database (HGMD, version 2024.4; https://www.hgmd.cf.ac.uk/ac/gene.php?gene=SGCA) lists 161 distinct *SGCA* variants reported in patients with LGMD R3. These variants are distributed across the entire gene, with exon 3 representing a notable mutational hotspot (Alonso‐Pérez et al. 2022).

Among the 39 cases genetically confirmed by NGS, *SGCA* emerged as the most frequently mutated gene, accounting for 10 patients in our cohort. Several distinct variants were identified, all in homozygous or compound heterozygous states, consistent with the autosomal recessive mode of transmission of LGMD R3. Most of these variants were absent from major public databases, including gnomAD and ClinVar, confirming their novelty and potential specificity to our population.

Notably, the c.956+1G>A splice‐site variant was identified in three unrelated families, each presenting with a clinical picture consistent with LGMD. The age of onset ranged from 3 to 10 years, and all affected individuals exhibited progressive proximal muscle weakness, gait difficulties, and markedly elevated CPK levels. Electromyography (EMG) consistently showed a myogenic pattern. One of the three patients underwent immunohistochemical analysis, which revealed a complete absence of α‐sarcoglycan expression, while β‐, γ‐, and δ‐sarcoglycans were preserved, supporting the implication of *SGCA*. Additionally, the variant affects a highly conserved canonical splice site, reinforcing its predicted deleterious effect. Given that this variant has not been reported in public databases and was previously reported only once by our department, it may represent a founder mutation specific to the Moroccan population (Rahmuni et al. 2024).

The c.411dup frameshift variant in the *SGCA* gene was identified in two unrelated female patients, both presenting with a proximal muscle weakness pattern, elevated CK levels, and myopathic findings on EMG. Age at onset differed between the two cases, with one developing symptoms at 3 years and the other at 7 years. Neither patient showed evidence of cardiac involvement.

One of the patients was referred at the age of 5 years and underwent immunohistochemical analysis prior to NGS testing, which revealed a complete absence of α‐sarcoglycan expression, while other sarcoglycans were preserved. The other patient was referred to our service at the age of 27 years, by which time she had already lost ambulation and was wheelchair‐bound, in line with literature reports describing loss of ambulation during the second decade of life in LGMD R3. She also presented with calf hypertrophy, a clinical feature frequently observed in this subtype (Sánchez Riera et al. 2021; Marsolier et al. 2017).

It has been reported in the literature that exon 3 of the *SGCA* gene represents a mutation hotspot, with a recurrent variant c.229C>T (Fernández‐Eulate et al. 2020). This recurrent mutation has been described in several populations, notably European and Brazilian, but to our knowledge, it has never been identified in the North African population. In our cohort, no patients harbored this variant. However, two other variants located within exon 3 (c.161delT and c.271G>A) were identified in two patients.

#### 4.2.3 LGMDR 1

LGMD R1 (OMIM #253600) is regarded as the most common form of recessive LGMD in many populations, representing approximately 30%–40% of reported cases (Balci et al. 2006). Variants in the *CAPN3* gene, located on chromosome 15q15.1–15.3, underlie the disease. The gene contains 24 exons spanning about 53 kb of genomic DNA and encodes calpain‐3 (p94), a muscle‐specific enzyme belonging to the family of calcium‐activated neutral proteases (Ono et al. 2016; Bardakov et al. 2025). The age of onset ranges from 2 to 50 years and is classified as early, typical, or late (Angelini et al. 2010). Childhood onset is generally linked to more severe progression (Fardeau et al. 1996), though not always predictive of severity (Gallardo et al. 2011). Severity may be influenced by ethnicity, mutation type, especially biallelic variants causing complete calpain‐3 loss (Barp et al. 2020) and sex, with women showing higher serum CK levels (Pollitt et al. 2001; Pathak et al. 2020). Interfamilial variability is marked, while intrafamilial variability is limited (Fardeau et al. 1996; Schessl et al. 2008). Three phenotypes, severe early onset, moderate, and mild late onset are distinguished (Fardeau et al. 1996). Calpainopathy is characterized by symmetrical limb muscle weakness and atrophy, occasionally with mild asymmetry, and frequent pseudohypertrophy of the gastrocnemius (Barp et al. 2020). Progressive joint contractures, most commonly in the ankles, hips, knees, and elbows, are a hallmark and may define an early contracture phenotype (Fardeau et al. 1996; Pollitt et al. 2001; Richard et al. 2016; Mercuri et al. 2005). Respiratory muscle involvement is typically late and mild, though severe impairment and rare early respiratory failure have been reported (Fardeau et al. 1996; Gallardo et al. 2011; Richard et al. 2016). Cardiac manifestations are usually benign, and significant dysfunction should raise suspicion for other LGMD subtypes (Richard et al. 2016; Okere et al. 2013; Verhaert et al. 2011). Among the seven patients carrying variants in the *CAPN3* gene (six females and one male), six were born to consanguineous parents. Five patients presented symptom onset between 12 and 15 years of age, while the remaining two developed symptoms later, at 24 and 33 years, respectively. This distribution is in line with the wide variability in age at onset described in the literature, although most of our cases fall within the typical onset period of adolescence and early adulthood. The first manifestations were exercise intolerance, difficulty climbing stairs, muscle pain, and gait disturbances. A positive Gowers' sign was observed in four patients, and calf hypertrophy was present in two cases. In addition, one patient developed tendon contractures and another presented with dorsal scoliosis. Notably, two patients lost ambulation at the ages of 15 and 19 years, respectively; both had siblings with similar symptomatology who also became non‐ambulant. These observations are consistent with the clinical spectrum of calpainopathy, while also underscoring the heterogeneity in disease progression.

A multicenter Chinese study published in 2020 analyzed the worldwide spectrum of *CAPN3* mutations in LGMD R1 and identified several risk exons. In the global patient dataset, the most frequently mutated exons were 1, 10, 5, 22, and 13, whereas in Chinese patients they were exons 1, 10, 13, 22, and 5. After adjusting for exon length, the highest mutation densities were observed in exons 15, 22, 21, 10, and 19. Pathogenic variants were distributed throughout the calpain‐3 protein, present in all calcium‐binding regions but absent from the protease active sites. Two recurrent mutations were reported: c.2120A>G, predominant in China, and c.550del, also described in European patients (Zhong et al. 2021). To our knowledge, calpainopathies have not been studied in Moroccan or North African populations. In our cohort, we identified a novel variant, c.2129G>A, in three unrelated patients, twice homozygous and once as a compound heterozygote with the known variant c.2242C>T, which was also found homozygous in another patient. Interestingly, the exons frequently mutated in previously published studies, as well as the recurrent mutations reported in Chinese or European populations, do not correspond to those observed in our series. These findings highlight c.2129G>A as a potentially population‐specific mutation and underscore the unique mutational spectrum of LGMD R1 in Moroccan patients, reinforcing the importance of studying underrepresented populations.

#### 4.2.4 LGMD R9

The *FKRP* gene, located on chromosome 19q13.3 and spanning about 12.5 kb, encodes fukutin‐related protein, a ribitol phosphate transferase required for proper glycosylation of α‐dystroglycan and its interaction with laminin in the extracellular matrix (Kanagawa and Toda 2017; Brockington et al. 2001). Pathogenic variants in the *FKRP* gene are associated with a wide clinical spectrum ranging from autosomal recessive LGMD R9 (Brockington 2001), to merosin‐deficient congenital muscular dystrophy type 1C (MDC1C) (Brockington et al. 2001), as well as the more severe phenotypes of Walker‐Warburg syndrome and Muscle‐Eye‐Brain disease (Beltran‐Valero De Bernabe 2004; Van Reeuwijk et al. 2010). Pathogenic and likely pathogenic variants in the *FKRP* gene were identified in three unrelated patients, all presenting with early disease onset, elevated CPK, and myogenic EMG changes, but without cognitive impairment or additional systemic involvement. Notably, we detected compound heterozygosity for c.1364C>A with either c.1012G>T or the novel variant c.1327G>A, as well as a homozygous variant: c.1016G>C. The recurrent variant c.1364C>A had previously been reported in a Tunisian cohort in 2004, where it was found in six patients at the homozygous state (Triki et al. 2004). Those patients presented with a much more severe phenotype, including congenital onset, inability to walk, intellectual disability, cerebellar abnormalities, and in some cases cardiac dysfunction. In contrast, our patients carrying c.1364C>A in a compound heterozygous state showed a considerably milder presentation, with preserved ambulation and no cognitive involvement. This difference may be explained by the allelic combination, as compound heterozygosity with another *FKRP* variant rather than homozygosity for c.1364C>A seems to attenuate disease severity. Taken together, and in line with their relatively mild phenotype compared to the Tunisian cohort, our patients can be classified as having LGMD R9. The recurrence of c.1364C>A in both Tunisian and Moroccan patients also raises the possibility that this mutation could be specific to North African populations. These findings expand the mutational spectrum of the gene by reporting c.1327G>A for the first time and further illustrate the variability of FKRP‐related disorders. Consistent with previous studies, our results reinforce the absence of a strict genotype–phenotype correlation in the *FKRP* gene, where even recurrent variants can lead to markedly divergent clinical outcomes.

#### 4.2.5 Other Genetic Findings and Diagnostic Reassessment

In addition to the results reported above, several variants were identified in other myopathy‐related genes in our cohort, and these findings are summarized in Table 3. Some of them had a significant impact on diagnostic orientation. In our study, an 8‐year‐old female patient referred for suspected LGMD was unexpectedly found to harbor a pathogenic variant in the *DMD* gene (*DMD*:c.10171C>T), which has already been reported in public databases as pathogenic and presented with a myopathic phenotype. She manifested early signs including fatigability both at rest and upon effort, as well as calf hypertrophy. This was a sporadic case, born from a non‐consanguineous marriage, and no echocardiographic evaluation was available at the time of study. Although females are usually asymptomatic carriers of X‐linked recessive diseases, symptomatic female carriers have been reported. For example, a 2014 study described six symptomatic females with *DMD* mutations who exhibited mild but progressive muscular weakness, elevated CK levels, and reduced dystrophin expression (Giliberto et al. 2014). In that study, symptoms were explained either by skewed X‐chromosome inactivation or by a translocation between the X chromosome and an autosome. More generally, expression of X‐linked recessive diseases in females can result from several mechanisms, including Turner syndrome (single X chromosome carrying the mutation), sex chromosome anomalies (e.g., XY karyotype), X‐autosome translocations, or skewed X‐inactivation. In our patient, while the *DMD* variant likely accounts for her symptoms, additional analyses, including X‐inactivation studies, karyotype or translocation analysis, segregation studies, and possibly muscle biopsy, are still required to confirm the diagnosis and clarify the underlying mechanism.

**TABLE 3:** Summary of other variants identified.

Gene	Inheritance	Variant (cDNA)	Protein change	Zygosity	N/R	Prediction (ACMG)	Detection method	Number of cases	Clinical comment
*COL6A1*	AR	c.98‐1G>C	p.?	Homozygous	N	Likely pathogenic	WES	2	Ullrich congenital muscular dystrophy 1A
		c.868G>A	p.Gly290Arg	Homozygous	R	Pathogenic	Targeted panel		
*COL6A2*	AR	c.838G>C	p.Gly280Arg	Homozygous	R	Pathogenic	WES	1	Bethlem myopathy 1B
*DYSF*	AR	c.5419C>T	p.Arg1807Trp	Homozygous	R	Pathogenic	WES	1	Compatible with LGMD R2
*SGCB*	AR	c.917_919del	p.Cys307del	Homozygous	N	Likely pathogenic	Targeted panel	1	Suggestive of LGMD R4
*SGCD*	AR	c.823C>T	p.Ser274Phe	Homozygous	N	VUS	WES	1	Further clinical correlation needed
*SGCG*	AR	c.525delT	p.Phe175fs	Compound heterozygous	R	Pathogenic	Sanger	1	Compatible with LGMD R5
		c.161C>A	p.Thr54Lys		R	VUS	WES		
*EMD*	XLR	c.399+1G>T	p.?	Hemizygous	R	Pathogenic	Targeted panel	1	Compatible with EDMD 1
*LMNA*	AD	c.1549_1550del	p.Gln517fs	Heterozygous	R	Pathogenic	Targeted panel	1	Suggestive of EDMD 2
*CRPPA*	AR	c.637A>G	p.Met213Val	Homozygous	N	Likely pathogenic	WES	1	Consistent with dystroglycanopathy
*MTMR14*	AD	c.721G>A	p.Asp241Asn	Heterozygous	R	VUS	WES	1	Requires further assessment
*HNRNPDL*	AD	c.98G>C	p.Arg33Pro	Heterozygous	R	VUS	WES	1	Atypical LGMD phenotype
*DMD*	XLR	c.10171C>T	p.Arg3391Ter	Heterozygous	R	Pathogenic	WES	1	Female carrier; incidental finding
*GAA*	AR	c.2173C>T	p.Arg725Trp	Compound heterozygous	R	Pathogenic	WES	1	Consistent with late‐onset Pompe disease
		c.716T>G	p.Leu239Arg		N	Likely pathogenic			
*DYNC1H1*	AD	c.3278T>C	p.Phe1093Ser	Heterozygous	R	Pathogenic	WES	1	Likely spinal muscular atrophy type
*MFN2*	AD	c.2258dup	p.Gln754fs	Heterozygous	R	Pathogenic	WES	1	Consistent with Charcot–Marie–Tooth disease, axonal, type 2A2A
*TTN*	AR	105946G>T	p.Glu3516Ter	Homozygous	N	Likely pathogenic	WES	1	Consistent with LGMD R10
*TOR1A*	AD	c.958A>G	p.Lys320Glu	Heterozygous	R	Pathogenic	WES	1	Requires further assessment
*MYBPC3*	AD	c.2221del	p.Ala741fs	Heterozygous	R	Pathogenic	WES	1	Cardiomyopathy
*POMGNT1*	AR	c.695_696insG	p.Ser233Phefs	Homozygous	N	Likely pathogenic	WES	1	Consistent with LGMD R15
*GGPS1*	AR	c.770T>G	p.Phe257Cys	Homozygous	R	Likely pathogenic	WES	1	Muscular dystrophy, congenital hearing loss, and ovarian insufficiency syndrome

Abbreviations: ACMG, American College of Medical Genetics and Genomics; AD, autosomal dominant; AR, autosomal recessive; EDMD, Emery–Dreifuss muscular dystrophy; LGMD R10, limb‐girdle muscular dystrophy, recessive 10; LGMD R15, limb‐girdle muscular dystrophy, recessive 15; LGMD R4, limb‐girdle muscular dystrophy, recessive 4; N, novel; R, reported; WES, whole‐exome sequencing; XLR, X‐linked recessive.

Another patient carried two compound heterozygous variants in the *GAA* gene, which encodes acid α‐glucosidase, the lysosomal enzyme deficient in Pompe disease and responsible for its pathogenesis. These included c.716 T>G, previously reported in public databases, and c.2173C>T, which to our knowledge has not been described before and is therefore considered novel. Pompe disease is an autosomal recessive disorder broadly divided into two clinical forms: the infantile and the late‐onset types. The infantile form manifests within the first months of life with generalized hypotonia, profound muscle weakness, respiratory distress, and hypertrophic cardiomyopathy, leading to death within the first year if untreated (Kishnani, Hwu, et al. 2006). By contrast, late‐onset Pompe disease presents with a more heterogeneous course. It is typically marked by a slowly progressive proximal muscle weakness, frequently accompanied by respiratory involvement and persistent hyperCKemia, while cardiac manifestations are generally absent (Schüller et al. 2012; Kishnani, Steiner, et al. 2006). Our patient was 13 years old, and she presented with myopathic signs, elevated CK and respiratory impairment, which is highly consistent with late‐onset Pompe disease. This case illustrates the importance of the genotype–phenotype correlation, as molecular results allowed us to refine the diagnosis initially suspected as LGMD. Pompe disease is a relatively frequent but still underestimated condition: many cases remain misdiagnosed, and in its infantile form, untreated patients often die before the age of one due to severe cardiomyopathy and respiratory failure. In contrast, late‐onset cases may remain unrecognized for years, being misclassified as other myopathies. Similar cases have been described in the literature, where patients initially suspected of having LGMD were later reclassified as Pompe disease following genetic testing (Johnson et al. 2017). These findings highlight the crucial role of molecular testing not only in confirming suspected diagnoses but also in redirecting them, an essential step when dealing with treatable conditions such as Pompe disease.

We also identified an 11‐year‐old patient, born to consanguineous parents, who presented with first symptoms at the age of 6, including proximal muscle weakness and calf hypertrophy. He was found to be a compound heterozygote for the recurrent frameshift variant *SGCG*: c.525delT and the missense variant *SGCG*:c.161C>A, the latter initially classified as a variant of uncertain significance (VUS). Muscle biopsy findings were consistent with a diagnosis of LGMD R5, further supporting the involvement of this second variant. Based on ACMG criteria, c.161C>A can be reclassified as likely pathogenic, as it fulfills PM3 (detected in trans with a known pathogenic variant) and PP1 (segregation with disease in affected relatives). Clinically, the patient had two brothers presenting with the same symptomatology, strengthening the evidence for pathogenicity of this allelic combination. Segregation analysis would be valuable to further confirm these findings.

As part of the diagnostic reassessment, particular attention was given to additional LGMD and EDMD subtypes previously reported in populations of similar ethnic background. Several subtypes of LGMD share genetic overlap with certain dystroglycanopathy forms of congenital muscular dystrophy, including the entity known as limb‐girdle muscular dystrophy‐dystroglycanopathy type C12 (OMIM 616094), which results from homozygous mutations in the *POMK* gene. A novel presentation of this condition was described in two siblings born to consanguineous Jordanian parents, who exhibited limb‐girdle congenital muscular dystrophy associated with cognitive impairment (Salih 2023; Di Costanzo et al. 2014). Likewise, numerous genes have been implicated in the etiology of Emery‐Dreifuss muscular dystrophy (EDMD). The fifth causative gene to be identified was *FHL1* (OMIM 300163), reported in 2009 in six unrelated families, including a Saudi family with five affected individuals and in an additional isolated case (Gueneau et al. 2009).

In our cohort, however, no pathogenic variants involving *POMK* or *FHL1* were detected. For EDMD, the two unrelated families identified in our series carried pathogenic variants in the major EDMD genes *EMD* and *LMNA*. Both families were analyzed using our customized targeted panel, which also includes *FHL1*, particularly since an X‐linked inheritance pattern was suspected in one of them which is consistent with variants in both *EMD* and *FHL1* genes. The absence of *POMK*‐related dystroglycanopathy in our cohort may be explained by our inclusion criteria, which focused on patients with symptom onset at or after 2 years of age, whereas many *POMK*‐associated phenotypes manifest congenitally or during early infancy.

It is also important to note that several patients in our cohort have not yet undergone NGS, and the genetic investigation of unresolved cases remains ongoing. Therefore, expanding NGS testing to these individuals may eventually uncover additional rare subtypes, although this remains challenging in our resource‐limited context.

The confirmation of variants by Sanger sequencing is an important step to validate NGS findings and exclude potential sequencing artefacts. When possible, segregation analyses can provide valuable information for variant reclassification, particularly for variants initially classified as VUS, according to ACMG criteria. The contribution of Sanger sequencing is therefore major for confirmation, segregation, and interpretation of variant pathogenicity, while clinical, ethical, and patient‐related considerations may influence the feasibility of these analyses.

## 5 Conclusion

This study provides the most comprehensive molecular overview of muscular dystrophies in the Moroccan population to date, combining first‐line routine testing and next‐generation sequencing approaches. Routine tests, including multiplex PCR for *DMD* and targeted Sanger sequencing of the recurrent *SGCG*:c.525delT variant, accounted for nearly half of the resolved cases, confirming their high diagnostic yield and continued relevance as front‐line strategies in our setting. NGS, applied through both targeted panels and exome sequencing, expanded the diagnostic spectrum by identifying pathogenic variants in *SGCA*, *CAPN3*, *FKRP*, and several other genes, thereby highlighting the marked genetic heterogeneity of muscular dystrophies in Morocco.

Importantly, recurrent variants were observed in *SGCA*, *CAPN3*, and *FKRP* genes, raising the possibility of population‐specific alleles that may be integrated into future diagnostic algorithms. While nearly half of cases remain unresolved, the integration of broader sequencing strategies with careful genotype–phenotype correlation is expected to improve diagnostic yield in the future. Overall, our findings emphasize the utility of a tiered testing approach adapted to resource‐limited contexts and provide a foundation for refining national diagnostic strategies for muscular dystrophies.

## Author Contributions

**Yasmina Rahmuni:** carried out the molecular genetic studies, including the targeted NGS gene panel and drafted the manuscript. **Ilham Ratbi:** performed the clinical genetic diagnosis, reviewed the manuscript, and supervised the study. **Jaber Lyahyai:** performed NGS data analysis. **Imane Cherkaoui Jaouad:** contributed to the clinical genetic studies; **Ourayna Batta:** contributed to the molecular genetic studies, including the targeted NGS gene panel; **Aziza Sbiti:** contributed to establishing the multiplex PCR assay and to molecular diagnostic activities. **Maryem Sahli:** contributed to the clinical and molecular genetic studies. **Omar Askander and Zineb Rchiad:** contributed to the whole‐exome sequencing. **Abdelaziz Sefiani:** conceptualization and supervision of the study.

## Funding

The authors have nothing to report.

## Ethics Statement

This study was approved by the Rabat ethics committee for Biomedical research (approval number: CERB‐37‐23).

## Consent

Informed consent was obtained from all individual participants (or their parents) included in the study.

## Conflicts of Interest

The authors declare no conflicts of interest.

## Data Availability

All data generated during this work are included in this article.
